# Evolution of genome-wide methylation profiling technologies

**DOI:** 10.1101/gr.278407.123

**Published:** 2025-04

**Authors:** Carolina Montano, Winston Timp

**Affiliations:** 1 Department of Biomedical Engineering, Johns Hopkins University, Baltimore, Maryland 21218, USA;; 2 Division of Human Genetics, Department of Pediatrics, Children's Hospital of Philadelphia, Philadelphia, Pennsylvania 19104, USA

## Abstract

In this mini-review, we explore the advancements in genome-wide DNA methylation profiling, tracing the evolution from traditional methods such as methylation arrays and whole-genome bisulfite sequencing to the cutting-edge single-molecule profiling enabled by long-read sequencing (LRS) technologies. We highlight how LRS is transforming clinical and translational research, particularly by its ability to simultaneously measure genetic and epigenetic information, providing a more comprehensive understanding of complex disease mechanisms. We discuss current challenges and future directions in the field, emphasizing the need for innovative computational tools and robust, reproducible approaches to fully harness the capabilities of LRS in molecular diagnostics.

## What is methylation?

Cytosine DNA methylation (5mC) involves the covalent modification of the fifth carbon atom of cytosine, typically in mammals in a CG dinucleotide context. This dinucleotide is depleted largely across the genome and found predominantly in clusters known as “CpG islands” (Bird 1986). Cytosine methylation is catalyzed by methyltransferases (e.g., DNMT1, DNMT3A) that transfer a methyl group from *S*-adenosyl-l-methionine to cytosine. Importantly, due to the palindromic nature of the CG dinucleotide, CG methylation can be retained after DNA replication by methylation of hemimethylated (i.e., one-strand methylated) DNA. While most mammalian cells exhibit only CG methylation, neurons, oocytes, and embryonic stem cells have shown DNA methylation (DNAm) in non-CG contexts, such as mCHG and mCHH, where H represents A, C, or T (Kinde et al. 2015). These are notably less mitotically active, presumably because such marks are more difficult to maintain with active replication.

Cytosine methylation plays a critical role in genomic imprinting, gene regulation, X-Chromosome inactivation (XCI), cellular differentiation, aging, and tumorigenesis. Cells have an extensive system of proteins that establish these methylation patterns through de novo methylation or demethylation, copy methylation patterns during DNA replication to sustain methylation levels, and read the DNAm states.

## How is methylation measured?

**Figure 1.**
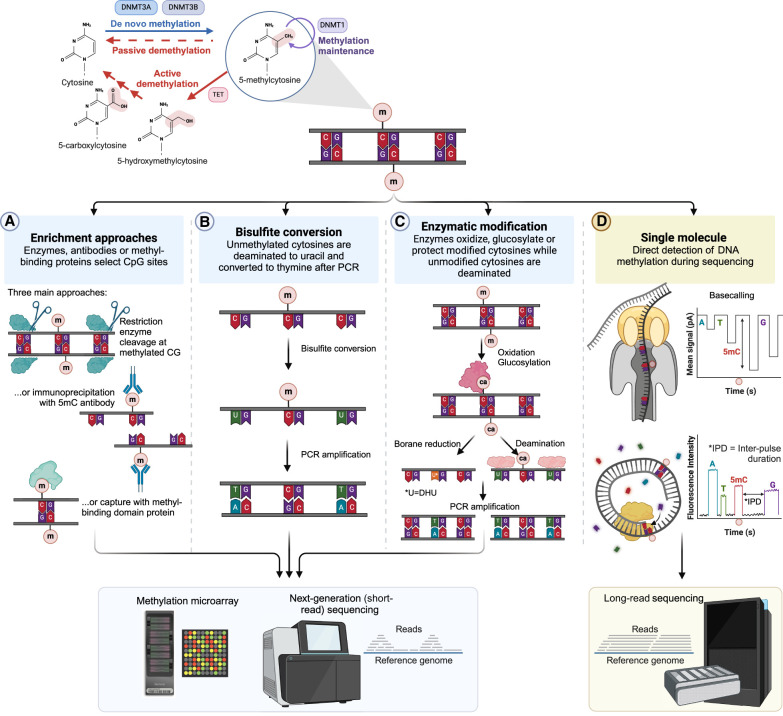
Methods to detect genome-wide 5mC. DNAm can be detected through enrichment, bisulfite conversion, enzymatic modification, or single-molecule approaches. (*A*) Enrichment methods use restriction enzymes or immunoprecipitation to separate methylated and unmethylated DNA. (*B*) Bisulfite treatment converts unmethylated cytosine to uracil, allowing differentiation between methylated and unmethylated DNA that can be assayed using microarrays or short-read sequencing. (*C*) Newer enzymatic methods use a series of proteins that oxidize and glycosylate modified cytosines, followed by deamination that is achieved either through specific enzymes or borane reduction. (*D*) Single-molecule methods directly measure DNAm as DNA passes through the nanopore (Oxford Nanopore, *top* panel) or as fluorescent nucleotides are incorporated into the growing strand by a polymerase (PacBio, *middle* panel). Created in BioRender. Montano, C. (2024). https://BioRender.com/i42h077.

Over the years, genome-wide DNAm profiling has evolved, progressing from methylation arrays to short-read whole-genome sequencing and, most recently, advancing into single-molecule profiling. These profiling methods can be broadly classified into enrichment or conversion methods. Enrichment methods (Fig. 1A) use either immunoprecipitation of methylated DNA (MeDIP) (Weber et al. 2005) or methylation-sensitive restriction enzymes (MSREs) (Khulan et al. 2006; Irizarry et al. 2008), which enrich methylated or unmethylated molecules based on size fractionation (Weber et al. 2005) followed by either quantitative polymerase chain reaction (qPCR), microarrays or sequencing. In contrast, bisulfite treatment of DNA, as developed by Frommer et al. (1992) and later optimized by Clark et al. (1994), enabled quantitative nucleotide resolution of methylation. It converts unmethylated cytosine to uracil by chemical deamination, while methylated DNA remains unconverted (Fig. 1B). After polymerase chain reaction (PCR), the methylated and unmethylated bases are distinguished as cytosine (C) to thymine (T) single nucleotide polymorphisms (SNPs).

The resulting data can be used to identify methylation “episignatures”—distinctive patterns of DNAm differences at specific CpG sites that are associated with pathogenic variants in particular genes (Grafodatskaya et al. 2013; Barbosa et al. 2018). Illumina's Infinium MethylationEPIC array, which targets nearly a million methylation sites, is the most widely used platform for this analysis. Microarray methylation data, e.g., The Cancer Genome Atlas (TCGA) Research Network and the International Cancer Research Consortium (ICGC), have facilitated the development of clinical-grade classifiers that leverage machine-learning episignatures for diagnosing brain tumors (Wu et al. 2022) and certain Mendelian disorders (Sadikovic et al. 2020). While clinical implementation requires careful standardization of analysis pipelines and reporting frameworks, leading laboratories now offer validated testing for over 90 genes and disorders (EpiSign). Despite their diagnostic value in enhancing the genetic testing yield for undiagnosed cases (Louis et al. 2021; LaFlamme et al. 2024), these arrays are not yet routinely used in constitutional molecular diagnostics due to additional costs and labor (Sadikovic et al. 2020).

The additional steps involved in array-based epigenomic profiling have restricted its use compared to larger-scale genomics studies. For instance, the largest population study to date using epigenomic profiling using Illumina Infinium microarrays involved 32,851 participants (Min et al. 2021), compared to the UK Biobank's exome sequencing of 454,787 individuals (Backman et al. 2021) and whole-genome sequencing of 150,119 individuals (Halldorsson et al. 2022).

## Whole-genome bisulfite sequencing

Alternatively, bisulfite-treated DNA can be used directly for sequencing instead of microarrays. This technique, known as whole-genome bisulfite sequencing (WGBS), is now considered the gold standard for detecting 5mC (Cokus et al. 2008; Lister et al. 2009). With the substantial drops in sequencing cost over the past 20 years, this method has come to dominate in smaller cohort sizes. However, WGBS has several limitations: the bisulfite conversion process can degrade DNA, reduce the complexity of the genome, and complicate the alignment process, particularly in repetitive regions (Karimzadeh et al. 2018).

Methods like TET-assisted pyridine borane sequencing (TAPS) (Liu et al. 2019), New England Biolabs Enzymatic Methyl-sequencing (NEB’s EM-seq) (Sun et al. 2021; Vaisvila et al. 2021), and biomodal's duet multiomics evoC (Füllgrabe et al. 2023) offer alternative approaches to WGBS by using enzymatic conversions and avoiding harsh bisulfite treatment which can degrade and fragment DNA (Fig. 1C). These methods use enzymes to oxidize, glucosylate, or protect modified cytosines, followed by deamination of unmodified cytosines. Compared to bisulfite methods, enzymatic methods provide larger library insert sizes, reduced DNA degradation, more uniform and less biased GC coverage, and even the ability to differentiate between 5mC and 5-hydroxymethylcytosine (5hmC) in some cases.

## Single molecule

Direct DNA sequencing using long-read sequencing (LRS) platforms like Oxford Nanopore Technologies (ONT) and Pacific Biosciences (PacBio) offers new opportunities to uncover novel genomic and epigenomic mechanisms implicated in disease. Sequencing reads generated by LRS not only detect single nucleotide variations (SNVs) and structural variations (SVs) but also probe previously uncharacterized repetitive regions and regions with atypical GC content, identify segmentally duplicated genes, and enable the phasing of reads (Logsdon et al. 2020). The phasing specifically allows for the assignment of haplotypes to pathogenic variants, enhancing diagnostic utility. LRS advancements enable the simultaneous measurement of DNAm profiles alongside genetic data, enhancing our understanding of methylation's role in diseases and its potential as a biomarker for Mendelian conditions (Fig. 1D; Flusberg et al. 2010; Simpson et al. 2017). But in contrast to microarray and short-read sequencing methods, DNAm data are inherently generated as a by-product of LRS, requiring no additional processing steps and thus facilitating its integration into existing genomic diagnostic workflows. Ongoing improvements in accuracy, throughput, and cost reduction further broaden LRS's applications across model and nonmodel organisms. With decreasing costs, LRS is now practical for generating comprehensive genomic and epigenomic profiles of patients. Instances of ultrarapid sequencing in critical care settings have demonstrated the effectiveness of same-day LRS workflows for clinical genetics, as shown by Gorzynski et al. (2022) and Goenka et al. (2022).

LRS technologies have now reached levels of accuracy, scalability, and cost-effectiveness that support their application in detecting variants across populations (De Coster et al. 2021). Major initiatives are demonstrating this capability at scale: deCODE has generated LRS data from 7179 Icelanders (Stefansson et al. 2024), while the Human Pangenome Reference Consortium (HPRC) has released 47 haplotype-resolved human genomes from diverse backgrounds (Liao et al. 2023). Standardizing the workflows, Kolmogorov et al. (2023) have developed a wet laboratory and computational workflow tailored to both cell lines and brain tissue samples. Multiple efforts are now generating crucial control data sets: the 1000 Genomes Project ONT Sequencing Consortium is producing LRS data from 1kGP samples to aid in filtering and prioritizing SNVs and SVs (Gustafson et al. 2024), while the All of Us (AoU) initiative has begun a long-read arm to sequence blood samples from Americans from diverse backgrounds, providing an essential population-level reference for comparing clinical samples. For phase I, they have sequenced 1027 individuals; phase 2 expands sequencing to 14,000 individuals and will incorporate methylation data, generating vast amounts of DNAm data as a “free” by-product of LRS. All of these efforts are generating vast amounts of DNAm data as a “free” by-product of LRS. However, researchers must ensure they conserve base modification information along with the sequencing data, as processing this methylation data requires additional computational tools. Luckily, this is aided and abetted by new standards of encoding methylation information directly into the alignment files (BAMs) as sequence tags.

## LRS is changing the landscape of molecular diagnostics

Molecular diagnosis is crucial for managing genetic diseases, offering confirmation, specialized care, risk-based management, informed reproductive choices, and, in some cases, personalized therapies. LRS provides a single assay that can simultaneous measure SNV, SV, copy number variation (CNV), and epigenetic alterations, all with a single sequencing run. With costs continuing to come down and the demonstrated enhanced diagnostic yield (Mastrorosa et al. 2023), LRS could provide a clear path forward. Here, we focus on diagnostic applications of haplotype-aware methylation analysis:

### Imprinting disorders

**Figure 2.**
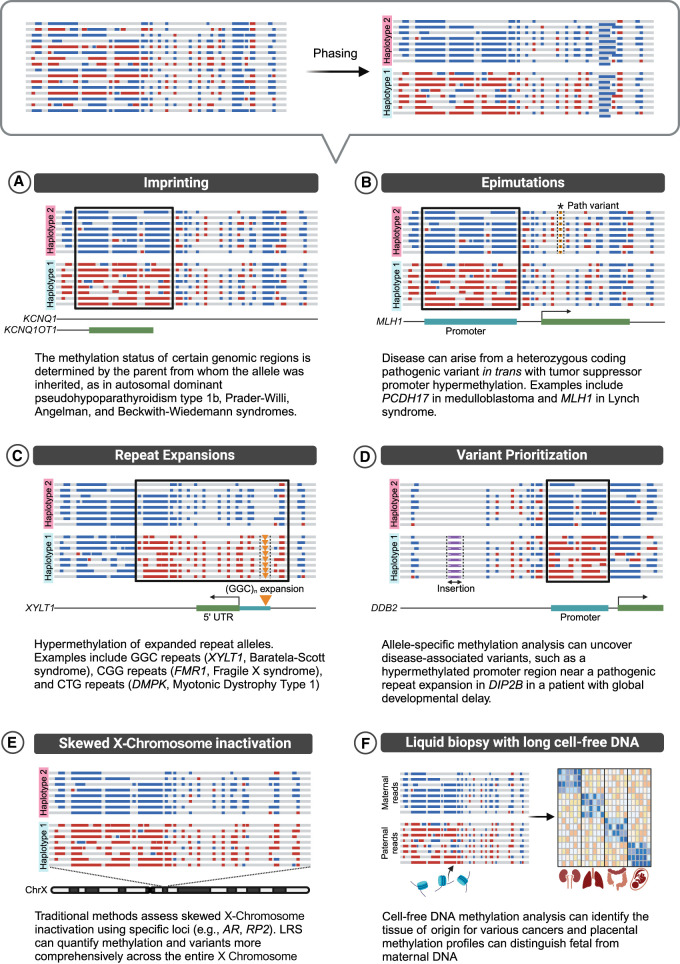
Diagnostic applications of haplotype-aware methylation analysis. Haplotype assignment after phasing significantly enhances the diagnostic utility of LRS. By enabling the simultaneous detection of SNVs, SVs, CNVs, and DNA modifications, it allows disease mechanisms that previously required separate specialized tests to be studied under a single, unified approach, such as imprinting (*A*), epimutations (*B*), and repeat expansions (*C*). It also facilitates variant prioritization (*D*), enables the investigation of skewed XCI where, instead of mixed inactivation, one haplotype is inactivated consistently (*E*), and can even be applied to study cell-free DNA (*F*). Created in BioRender. Montano, C. (2024). https://BioRender.com/h78t893.

Genomic imprinting refers to the parent-of-origin specific expression of genes; the classic example is the *H19/IGF2* locus with paternal expression of *IGF2* while the maternal copy is silenced (Monk et al. 2019). This is often regulated by epigenetic marks, including DNAm and histone posttranslational modifications. In the loss of imprinting disorders, either the active allele is silenced or the normally silent allele is abnormally activated (Eggermann et al. 2015). The underlying molecular defects are diverse, including pathogenic variants in imprinted genes, CNVs, uniparental disomy (UPD), and aberrant methylation over imprinting control regions. Traditionally, diagnosing these disorders required a stepwise combination of methylation (with methylation-specific PCR and MLPA), CNV (with oligo-SNP arrays and MLPA), single gene and microsatellite analysis, and often parental testing for variant phasing. While methylation-sensitive MLPA (MS-MLPA) is now the first-tier test, it is typically limited to single-locus analysis and cannot differentiate between UPD and imprinting defects, requiring additional assays (Grafodatskaya et al. 2017). In contrast, LRS provides a comprehensive solution by simultaneously detecting all types of genetic and epigenetic variation, phasing variants, and distinguishing maternal and paternal haplotypes based on methylation patterns at imprinted loci (Fig. 2A; Paschal et al. 2025). This capability is crucial for identifying multilocus imprinting disturbance (MLID), a condition that single-locus tests would miss (Sanchez-Delgado et al. 2016). Accurate molecular diagnosis using LRS is vital not only for clinical management but also for monitoring tumor risk associated with some imprinting disorders.

Miller et al. (2022) were among the first to demonstrate the utility of targeted LRS in investigating imprinted loci. They studied a family with autosomal dominant pseudohypoparathyroidism type 1b (PHP-1b) demonstrating the loss of methylation in *GNAS* was due to a maternally inherited retrotransposon insertion, highlighting the ability to detect both structural variants and methylation changes in a single assay. Yamada et al. (2023) further explored the diagnostic potential of targeted LRS in Prader–Willi syndrome (PWS) and Angelman syndrome (AS), two disorders caused by aberrant imprinting in the 15q11.2–q13 region (Yamada et al. 2023). They detected abnormal methylation patterns in the *SNRPN* gene, crucial for differentiating between PWS and AS, while simultaneously identifying the underlying pathogenic mechanisms, including uniparental isodisomy, in a single assay. A larger study by Paschal et al. (2025) extended the application of LRS to whole-genome analysis in a larger cohort of individuals with known PWS or AS. They found 100% concordance with standard clinical testing and could even phase the 15q11–q31.1 region to measure the likely parent of origin of *UBE3A* variants without parental samples. To give an easily interpretable report, Bækgaard et al. (2024) recently developed a tool for analyzing and reporting on 14 imprinted regions. They demonstrated this on samples from Beckwith–Wiedemann, Angelman, and Prader–Willi syndromes. While these studies are promising, further validation of LRS is needed to establish minimum coverage requirements, sensitivity, and specificity in other imprinted regions and for mosaic alterations for clinical applications.

### Epimutations

In genomic diagnostics, it is crucial to determine if variants occur in *cis* (same chromosome) or in *trans* (opposite chromosomes). This is particularly valuable for classifying variants according to ACMG guidelines, where detecting variants in *trans* provides stronger evidence of pathogenicity (Richards et al. 2015). In the case of recessive disorders, where two malfunctioning gene copies are required to cause disease, knowing whether two heterozygous variants are in *cis* or *trans* helps determine if both variants are harmful. For example, a variant in *trans* with a pathogenic variant can be considered as evidence of pathogenicity, whereas if this variant is found in *cis*, it is most likely benign. In contrast, for dominant disorders, where a single pathogenic variant is sufficient to cause disease, the presence of a second variant in *trans* with the pathogenic variant typically indicates benign impact. Traditionally, this required testing parental (or offspring) samples to establish variant inheritance and infer phasing, which can be challenging when familial samples are unavailable. LRS simplifies this process by directly covering both variants in a single haplotype-resolved read, allowing immediate *cis*/*trans* determination (Fig. 2B; Conlin et al. 2022).

The unique combination of phasing and methylation detection offered by LRS enables the identification of epimutations. While the role of epimutations in disease has been recognized for decades, their identification previously required separate, specialized testing (Hitchins et al. 2007). Most epimutations result in epigenetic silencing, frequently through promoter hypermethylation, mimicking the effects of a loss-of-function coding variant in the opposite allele. For example, promoter hypermethylation can serve as the “second hit” in tumors with heterozygous germline variants (Hitchins 2015). Rausch et al. (2023) demonstrated promoter methylation of the tumor suppressor *PCDH17* in medulloblastoma using LRS, underscoring the importance of LRS in elucidating the complex interplay of genetic and epigenetic factors in cancer development. In cancer predisposition disorders like Lynch syndrome, constitutional *MLH1* promoter hypermethylation acts as a “first hit,” requiring only a somatic “second hit” for tumor development. Nakamura et al. (2024) used LRS to evaluate patients with suspected hereditary tumor predisposition syndromes for allele-specific methylation differences, identifying constitutional *MLH1* promoter hypermethylation in a patient with Lynch syndrome after previous uninformative genetic testing. Similarly, O'Neill et al. used LRS to confirm Lynch syndrome in a patient by detecting constitutional *MLH1* promoter methylation in blood. Somatic loss of heterozygosity (LOH) in the patient's tumor led to the loss of the wild-type *MLH1* allele, leaving only the methylated allele (O'Neill et al. 2024).

### Repeat expansion disorders

LRS represents a significant advancement in diagnosing nucleotide repeat expansion disorders, which are notoriously difficult to identify and require multiple tests to characterize fully. Though PCR-based methods, such as genotyping, repeat-primed, and methylation-specific assays, have largely replaced the need for Southern blotting, they still need to be designed specifically for each repeat (Spector et al. 2021). However, these assays are technically challenging due to high GC content, repeat size polymorphism, and repeat sequence homology, requiring careful controls and often challenging to measure extreme length values (e.g., >250 CGG for *FMR1* repeats) (Chen et al. 2011). While genotyping software has facilitated the simultaneous detection of multiple tandem repeats using short reads, the repeat lengths require sufficient coverage at the locus and are estimated rather than precisely measured (Dolzhenko et al. 2017; Ibañez et al. 2022). In contrast, LRS provides not just length measurements as capillary electrophoresis does but also methylation and sequencing information (Fig. 2C). LRS data identifies polymorphic repeat counts—even over large and complex expansions that elude standard molecular testing—as well as methylation and even sequence variations within the repeats, all from one assay. This includes detecting repeat interruptions that stabilize alleles and protect against further expansion, allele-specific DNAm, and somatic mosaicism, making LRS a powerful tool for uncovering the epigenetic regulation of such repeats.

This comprehensive approach has not only improved the diagnosis of known repeat expansion disorders but has also contributed to the discovery of novel ones (Gall-Duncan et al. 2022). In Baratela–Scott syndrome, commonly caused by the expansion and methylation of GGC repeats in *XYLT1*, LRS identified methylation of the haplotype containing the permutation allele in some reads from a proband's mother (Miller et al. 2021). In Fragile X syndrome (FXS), expanded (>200) CGG repeats trigger hypermethylation of the X-linked *FMR1* promoter, resulting in gene silencing (Hunter et al. 1998). Notably, hypermethylation and repeat size do not always correlate perfectly, as some individuals with full mutation alleles lack promoter methylation and exhibit milder phenotypes (Tak et al. 2024). Conversely, we encounter premutation (∼55–200) alleles, which pose a risk of increasing in length during reproduction but are not yet at the disease threshold. These alleles often have altered methylation, challenging to measure with methylation-sensitive restriction-based assays, but which has shown an association between repeat length, methylation percentage, and phenotype severity (Pretto et al. 2014). Stevanovski et al. (2022) further validated this approach, demonstrating hypermethylation in both full mutation and premutation carriers. Similarly, Rasmussen et al. (2022) discovered that in patients with Myotonic Dystrophy Type 1 (DM1), hypermethylation of CTG repeats was observed only in the full-penetrance allele of *DMPK* (with the shortest allele detected in the study having 142 repeats). In these cases, hypermethylation of the expanded allele was more evident when allele-specific analysis was performed.

### Variant prioritization in rare disease

Allele-specific methylation analysis is also helpful in identifying and prioritizing previously missed SVs associated with methylation changes (Fig. 2D). In a large rare disease cohort, Cheung et al. (2023) discovered a hypermethylated region adjacent to a previously missed pathogenic repeat expansion in the *DIP2B* gene in a proband with global developmental delay. They also identified other genetic variations (insertions, SNVs, SVs, duplications, deletions) occurring in *cis* with proximal regulatory element hypermethylation and predicted to disrupt regulatory element activity.

### X-Chromosome inactivation

Allele-specific methylation is a critical mechanism in XCI, a dosage compensation process ensuring a single active X Chromosome in both XY and XX individuals (Lyon 1962). In typical XX individuals, one X is randomly silenced in each cell during development, with methylation maintaining gene repression on the inactive X (Xi). This random inactivation creates a mosaic of cells with either maternal or paternal X activation, and distinguishing the active X (Xa) and Xi in this mosaic cannot be done using SNPs alone. The gold standard method uses MSREs and PCR to assess methylation at the androgen receptor (*AR*) (Allen et al. 1992) and the RP2 activator of ARL3 GTPase (*RP2*) gene (Machado et al. 2014), utilizing their polymorphic repeats for parental allele identification (Caylor 2023). The ratio of inactivation between the two alleles can determine whether XCI is random (balanced) or skewed (nonrandom) (Amos-Landgraf et al. 2006). Skewed XCI can arise from various factors, including cell selection bias in carriers of X-linked pathogenic variants (Migeon 2020), secondary selection in X Chromosome rearrangements, or clonal populations in cancer, and can be informative for genetic counseling and testing (Amos-Landgraf et al. 2006).

We can quantify the entire X Chromosome with long reads measuring the level of methylation and genomic variants to gain allele-specific information (Fig. 2E; Lee et al. 2020). This provides a far more quantitative and comprehensive view than targeting a few genes (Johansson et al. 2023), enabling investigation of severely skewed inactivation cases, potentially uncovering previously missed X-linked pathogenic variants or X Chromosome rearrangements. This can include genes subject to or escaping XCI, satellites (Gershman et al. 2022), or focusing on the most informative CpG islands (Gustafson et al. 2024). LRS allows direct detection of repeat counts combined with methylation over *AR* or *RP2* CpG islands (Johansson et al. 2023) or even using chromatin accessibility (Lee et al. 2020; Vollger et al. 2025).

### Long cell-free DNA

Cell-free DNA (cfDNA) fragments circulating in bodily fluids like plasma and urine carry unique methylation patterns, acting as molecular fingerprints that reveal their cellular origins. Scientists have developed highly specific “liquid biopsies,” noninvasive biomarkers that identify the DNA's tissue or cell-type source by analyzing cfDNA methylation patterns (Yu et al. 2023a). This has led to applications in prenatal screening, where the methylation profile of fetal cfDNA correlates with placental methylation, allowing for the differentiation of maternal from fetal cfDNA without depending on genotypic information (Sun et al. 2015). Methylation patterns in circulating tumor DNA (ctDNA), analyzed through unique methylation signatures, are also emerging as powerful tools for cancer detection, enabling researchers to pinpoint the origin of a tumor, determine tumor burden, and even identify specific cancer types. Historically, this was done by examining differentially methylated loci using microarrays (Moss et al. 2018) or bisulfite sequencing (Guo et al. 2017) within the cfDNA, which offer superior accuracy compared to traditional methods relying on SNV. Multiple studies have revealed the predominance of tumor-derived DNA in the cfDNA pool of various solid cancers and have demonstrated the utility of methylation for estimating tumor load and identifying the tissue of origin across a wide range of cancer types (Guo et al. 2017; Moss et al. 2018; Liu et al. 2020). Enrichment strategies like cfMeDIP have been used to focus methylation profiling on cfDNA to establish cancer-specific patterns for early detection and diagnosis (Shen et al. 2018).

Although most cfDNA studies have focused on shorter sequences, analyzing longer cfDNA fragments with LRS offers distinct advantages due to the increased number of CpG sites, improving the accuracy of tissue-of-origin determination (Fig. 2F). This enhanced analysis has shown promising results in diverse applications, including identifying the parental origin of fetal cfDNA (Yu et al. 2021), determining the tissue of origin in various cancers (Choy et al. 2022; Lau et al. 2023), and monitoring tumor burden (Katsman et al. 2022). Analysis of tissue origin using single-molecule methylation patterns demonstrated similar performance on ONT and PacBio platforms (Yu et al. 2023b). Finally, even the size of cfDNA can be telling—studies have shown differences in cfDNA length—e.g., from normal to cancer samples (Cristiano et al. 2019).

## Biological and technical challenges in methylation analysis using LRS

Though the development and recent price drops in LRS technology provide unique opportunities, they also present unique challenges. With this new potential wealth of genomics data, one has to be careful to consider the epigenetic setting of samples for experimental design and interpretation

### Epigenetic experimental design

While genomic studies can reveal identical pathogenic variants in the germline from any sample, epigenomics studies require more attention to the sample source, as the epigenome is highly dynamic with substantial cell-type and tissue specificity (Laird 2010), particularly around noncoding regions of the genome (Kerr et al. 2023). Though large-scale genomic studies will indeed include epigenetic data, this data will likely be confined to easily accessible sample types, e.g., blood and saliva.

Tissue-specific methylation profiles are often the strongest epigenetic signature observed, even above disease or individual-specific epigenetic differences. The most significant methylation differences often occur between distinct tissues within an individual (Rakyan et al. 2008; Lokk et al. 2014; Loyfer et al. 2023). Even within the same tissue, cell heterogeneity and varying cell-type proportions can profoundly influence the observed epigenetic patterns, potentially leading to an underestimation of DNAm (Houseman et al. 2012; Guintivano et al. 2013; Jaffe and Irizarry 2014).

This fundamental tissue and cellular heterogeneity necessitate careful experimental design for epigenomic studies. For instance, LRS analysis of fibroblasts and whole blood from the same individual might identify identical pathogenic variants, yet their respective epigenomic signatures, and even disease-specific differences, will likely differ substantially. Tissue-specific data, as in a recent NIH CARD study (Kolmogorov et al. 2023), can offer valuable insights. But even in this case, the heterogeneity of brain tissue complicates the epigenetic analysis of shifts within specific cell populations, such as neurons. Other studies (Montaño et al. 2013; Rizzardi et al. 2019) have demonstrated that measuring more subtle epigenetic differences requires sorting of specific cell types. Researchers must establish comprehensive baseline epigenetic profiles of relevant tissues from unaffected individuals. Disease studies should then use biological replicates matched as closely as possible for age, sex, and tissue composition to these baseline samples. Tissue samples should either be sorted into specific cell populations or, at minimum, undergo careful profiling to determine their cellular composition, as cellular heterogeneity between samples can mask subtle but important epigenetic changes.

This underscores the importance of validating tissue-specific epigenetic signatures for each condition under investigation. Beyond tissue type, factors such as age, sex chromosome complement, and environmental exposures (e.g., smoking) can also significantly influence the epigenome (Michels et al. 2013). Consequently, robust experimental design for epigenomic studies requires large, age- and sex-matched negative control cohorts (Chater-Diehl et al. 2021), unlike the traditional parent-proband trio analysis typically used in genetic studies.

### Technical challenges

Though LRS is maturing as a platform, there are still significant growing pains. Differences in methylation calls between ONT and PacBio, chemistry versions, and methylation calling algorithms require careful consideration when comparing data across platforms (Genner et al. 2024; Sigurpalsdottir et al. 2024). Updates to the software and pore chemistry can also cause shifts—usually small ones, but shifts which may be important when comparing results within large cohorts. This is a significant concern when compared to the relative stability of bisulfite-based sequencing or microarray methods. Batch effect correction between chemistries and methods is necessary to compare technologies, chemistries, and basecallers.

While batch effects are well-studied in methylation arrays with established quality control (QC) pipelines using methods like surrogate variable analysis (SVA) and remove unwanted variation (RUV), standard pipelines for sequencing methylation data, especially LRS methods, still require development and benchmarking. To account for this currently, one needs to use purposeful study design (sample randomization, control samples) and QC metrics like principal component analysis (PCA) on the resulting data. Current work using resources like GIAB and 1000 Genomes data sets as benchmarking standards could result in the establishment of strong methods here, though comprehensive validation across methylation calling methods is still needed. Internal controls such as expected allele-specific methylation patterns or methylation state at housekeeping genes may be useful to normalizing methylation calls across different technologies and runs.

Furthermore, LRS native methylation methods, rather than providing a C or a T call as bisulfite sequencing does, give a probability score that the base is methylated. These scores can be thresholded and generally show high concordance with traditional methylation calling, but there may be biases in methylation calls depending on the local sequence context. To account for technical variability, it is crucial to define sequencing metrics and depth cutoffs for calling modification events or, at the very least, report these details in the methods section. Unfortunately, established tools for correcting batch effects in LRS methylation data are currently lacking (Kong et al. 2023).

Cancer and disorders with somatic mosaicism present unique challenges, requiring specialized analysis tools to address issues like variant allele frequency thresholds to detect cellular mosaicism, intratumor heterogeneity, normal cell contamination, and variations in tumor methylation levels. To overcome some of these obstacles, targeted sequencing of specific regions using Cas9-targeted sequencing (Tsai et al. 2017; Höijer et al. 2018; Gilpatrick et al. 2020), adaptive sampling (Miller et al. 2021), or hybridization capture (Mahmoud et al. 2024) has been used to isolate and analyze specific genomic regions of interest at higher coverage depth, akin to deep high-throughput sequencing panels. While standard methylation detection resolution is limited to 1/coverage levels (∼5% for an average 30× sequencing run), Ziller et al. (2015) demonstrated that even lower per-sample coverage (5–15×) can be sufficient to detect regional methylation differences between groups, especially when using more replicates.

### Data storage challenges

The storage and retention of raw sequencing data presents significant challenges for LRS methylation analysis. While raw signal files contain valuable information that enables rebasecalling with improved algorithms, their size makes long-term storage impractical. For example, ONT POD5 files from a single PromethION flow cell can exceed 1TB. This mirrors the historical practice of retaining Illumina BCL files, which long since have been discarded immediately after runs; the cost-benefit analysis increasingly favors resequencing over maintaining such large data archives. PacBio presents similar considerations. Kinetics and subreads are typically no longer stored directly due to the large file sizes from PacBio.

For methylation analysis specifically, the key decision point comes during basecalling. Advancements in BAM file specifications now allow for efficient storage of modification information directly within a BAM file itself using modification call (MM) and its associated probability (ML) tags for downstream analysis. This approach results in files ∼100 GB in size—a significant but manageable footprint. While traditional FASTQ files remain a mainstay of sequencing workflows, they strip out this crucial methylation information, making them unsuitable for methylation analysis despite their smaller size.

## Analysis of LRS methylation data presents unique opportunities

Analyzing DNAm data from LRS demands innovative computational and statistical approaches. Though one *can* take the methylation frequency files generated from pileups of methylation calls from LRS data and process them the same way one would a WGBS data set or even look at individual CpG methylation frequency like a microarray, this is throwing away some of the novelty of LRS data.

LRS has two significant virtues that are neglected by conventional analysis pipelines. First, it is a single molecule. Each LRS read represents one molecule from one allele from one cell in a sample. Typically, there is no amplification, so the measurements are a sampling of the molecules present in the DNA mix. The methylation patterns along the molecule can be phased along with the genotype to produce allele-specific methylation information—allowing us to interrogate methylation associated with specific alleles. It can even be phased to group with specific somatic mutations, allowing us to observe genomic and epigenomic interactions.

We lack robust statistical or analytical tools that identify loci with different methylation between germline alleles or between mutated and reference molecular groups. The use of simple Fisher tests to compare methylation levels is problematic, as single CpG analysis is susceptible to noise, which could lead to false conclusions. To address this, smoothing techniques (Hansen et al. 2012) are often used to exploit the natural correlation between adjacent CpGs, which helps reduce noise and improve the detection of differentially methylated regions (DMRs), especially in areas with nonuniform methylation.

These comethylation patterns, known as “methylation haplotype blocks” (Guo et al. 2017), are valuable for identifying tissue- and cell-specific signatures, facilitating cell-of-origin deconvolution in bulk tissue (Loyfer et al. 2023; Unterman et al. 2024). Although this approach can work with short-read sequencing, longer reads significantly enhance the likelihood of capturing cell-type-specific DMRs, thereby improving the effectiveness of deconvolution. However, identifying allele-specific DMRs requires more sophisticated methods.

However, there remains a need to establish appropriate thresholds for methylation frequency and region size while also developing haplotype-aware tools for novel DMR discovery. The inherent noise in methylation data, combined with reduced coverage due to haplotype splitting, remains a challenge for clinical applications. Moreover, linking haplotypes across multiple samples to define the *same* haplotype group is particularly challenging, and may require graph genome approaches or *k*-mer analyses to effectively connect haplotypes across larger data sets (Liao et al. 2023).

We can also use the single molecules to measure epigenetic and genetic interactions *along* a molecule. Consider that the length of LRS reads will often enable simultaneous measurement on a single molecule of enhancers and promoters, which are typically too distal to directly measure with short-read sequencing. This capability allows researchers to capture the regulatory landscape holistically, linking regulatory elements with their target genes on the same read. For instance, Nanopore sequencing has been used to phase enhancers and promoters, revealing allele-specific regulatory interactions that are crucial for understanding gene expression regulation and epigenetic modifications (Gigante et al. 2019). Additionally, this approach facilitates the study of chromatin interactions and the 3D genome architecture, providing insights into how distant regulatory elements come together to control gene activity (Lee et al. 2020; Zhong et al. 2023). Methods like nanoNOMe (Lee et al. 2020), Fiber-seq (Stergachis et al. 2020), DiMeLo-seq (Altemose et al. 2022), and SAMOSA-tag (Nanda et al. 2024) use methyltransferase enzymes to encode epigenetic information into DNA molecules, enabling LRS to simultaneously map chromatin accessibility, protein binding, and DNA modifications at single-molecule resolution. Such detailed mapping of regulatory landscapes is essential for identifying the mechanisms underlying complex traits and diseases and improving the accuracy of genomic and epigenomic annotations (Akbari et al. 2021).

Methylation deconvolution of individual reads could harness the single-molecule resolution of LRS to disentangle complex methylation patterns within heterogeneous samples. By analyzing methylation signals at the level of individual molecules, it could be possible to identify cell-type-specific methylation signatures and quantify allele-specific methylation variations. Such methods could leverage the read length of LRS to capture contiguous methylation states across entire genomic regions, potentially enabling the precise reconstruction of methylation landscapes often obscured in bulk analyses. Advanced computational tools are required to interpret these high-resolution methylation profiles accurately, which could offer new insights into epigenetic regulation and disease mechanisms.

Improved mappability through repeats and other hard-to-map regions is a significant advantage of LRS technologies (Ebbert et al. 2018; Giesselmann et al. 2019; Stevanovski et al. 2022; Dolzhenko et al. 2024). LRS platforms, such as ONT and PacBio, produce reads that span thousands of base pairs, allowing for the accurate sequencing of complex regions like tandem repeats, segmental duplications, and regions with low SNP density. Unlike short-read sequencing, which struggles with these regions, LRS provides a clearer and more comprehensive view of the genome. This capability is particularly valuable for detecting variations within repeats, such as those found in repeat expansion disorders, and for achieving precise haplotype phasing. Enhanced mappability improves variant detection and epigenetic profiling, facilitating a deeper understanding of genetic and epigenetic mechanisms underlying various diseases.

Accurate haplotype assignment and phasing tools continue to evolve, combining both genetic and epigenetic data. Tools like NanoMethPhase (Akbari et al. 2021) (for ONT), PRINCESS (Mahmoud et al. 2021) (for both ONT and PacBio), and ccsmeth (Ni et al. 2023) (for PacBio) leverage SNPs and methylation for phasing, enabling accurate, haplotype-resolved variant and methylation calls. While these approaches face challenges in heterogeneous tissue samples with different methyatlion profiles, they also could offer unique advantages, particularly in determining haplotype parent-of-origin without requiring trio sequencing data. Tools like MethPhaser (Fu et al. 2024) attempt to extend phase blocks using methylation data, though improvements may be modest in regions with few allele-specific methylation sites.

The potential of LRS remains constrained by the need for specialized tools and methodologies, particularly those focused on haplotype-aware analysis and noise reduction. Both standardization and benchmarking are essential for LRS to become a routine clinical tool. Although core technologies and algorithms are steadily improving the accuracy of LRS data, clinical laboratories require stable workflows to comply with stringent regulatory requirements. Additionally the current reliance on high-quality DNA, often obtained from frozen blood samples, presents logistical challenges for clinics without freezer storage. Other tissue specimens (e.g., biopsies) may also not offer the micrograms required for LRS methods. Thus, validating LRS in more readily accessible samples, such as saliva or buccal tissue, is crucial for broader clinical adoption.

## References

[GR278407MONC1] Akbari V, Garant J-M, O'Neill K, Pandoh P, Moore R, Marra MA, Hirst M, Jones SJM. 2021. Megabase-scale methylation phasing using nanopore long reads and NanoMethPhase. Genome Biol 22: 68. 10.1186/s13059-021-02283-5 33618748 PMC7898412

[GR278407MONC2] Allen RC, Zoghbi HY, Moseley AB, Rosenblatt HM, Belmont JW. 1992. Methylation of *Hpa*II and *Hha*I sites near the polymorphic CAG repeat in the human androgen-receptor gene correlates with X chromosome inactivation. Am J Hum Genet 51: 1229. 1281384 PMC1682906

[GR278407MONC3] Altemose N, Maslan A, Smith OK, Sundararajan K, Brown RR, Mishra R, Detweiler AM, Neff N, Miga KH, Straight AF, 2022. DiMeLo-seq: a long-read, single-molecule method for mapping protein-DNA interactions genome wide. Nat Methods 19: 711–723. 10.1038/s41592-022-01475-6 35396487 PMC9189060

[GR278407MONC4] Amos-Landgraf JM, Cottle A, Plenge RM, Friez M, Schwartz CE, Longshore J, Willard HF. 2006. X chromosome-inactivation patterns of 1,005 phenotypically unaffected females. Am J Hum Genet 79: 493–499. 10.1086/507565 16909387 PMC1559535

[GR278407MONC5] Backman JD, Li AH, Marcketta A, Sun D, Mbatchou J, Kessler MD, Benner C, Liu D, Locke AE, Balasubramanian S, 2021. Exome sequencing and analysis of 454,787 UK Biobank participants. Nature 599: 628–634. 10.1038/s41586-021-04103-z 34662886 PMC8596853

[GR278407MONC6] Bækgaard CH, Lester EB, Møller-Larsen S, Lauridsen MF, Larsen MJ. 2024. Nanoimprint: a DNA methylation tool for clinical interpretation and diagnosis of common imprinting disorders using nanopore long-read sequencing. Ann Hum Genet 88: 392–398. 10.1111/ahg.12556 38690755

[GR278407MONC7] Barbosa M, Joshi RS, Garg P, Martin-Trujillo A, Patel N, Jadhav B, Watson CT, Gibson W, Chetnik K, Tessereau C, 2018. Identification of rare de novo epigenetic variations in congenital disorders. Nat Commun 9: 2064. 10.1038/s41467-018-04540-x 29802345 PMC5970273

[GR278407MONC8] Bird AP. 1986. CpG-rich islands and the function of DNA methylation. Nature 321: 209–213. 10.1038/321209a0 2423876

[GR278407MONC9] Caylor RC. 2023. Nonrandom X chromosome inactivation detection. Curr Protoc 3: e748. 10.1002/cpz1.748 37074091

[GR278407MONC10] Chater-Diehl E, Goodman SJ, Cytrynbaum C, Turinsky AL, Choufani S, Weksberg R. 2021. Anatomy of DNA methylation signatures: emerging insights and applications. Am J Hum Genet 108: 1359–1366. 10.1016/j.ajhg.2021.06.015 34297908 PMC8387466

[GR278407MONC11] Chen L, Hadd A, Sah S, Houghton JF, Filipovic-Sadic S, Zhang W, Hagerman PJ, Tassone F, Latham GJ. 2011. High-resolution methylation polymerase chain reaction for fragile X analysis: evidence for novel *FMR1* methylation patterns undetected in Southern blot analyses. Genet Med 13: 528–538. 10.1097/GIM.0b013e31820a780f 21430544 PMC4043840

[GR278407MONC12] Cheung WA, Johnson AF, Rowell WJ, Farrow E, Hall R, Cohen ASA, Means JC, Zion TN, Portik DM, Saunders CT, 2023. Direct haplotype- resolved 5-base HiFi sequencing for genome-wide profiling of hypermethylation outliers in a rare disease cohort. Nat Commun 14: 3090. 10.1038/s41467-023-38782-1 37248219 PMC10226990

[GR278407MONC13] Choy LYL, Peng W, Jiang P, Cheng SH, Yu SCY, Shang H, Olivia Tse OY, Wong J, Wong VWS, Wong GLH, 2022. Single-molecule sequencing enables long cell-free DNA detection and direct methylation analysis for cancer patients. Clin Chem 68: 1151–1163. 10.1093/clinchem/hvac086 35587130

[GR278407MONC14] Clark SJ, Harrison J, Paul CL, Frommer M. 1994. High sensitivity mapping of methylated cytosines. Nucleic Acids Res 22: 2990–2997. 10.1093/nar/22.15.2990 8065911 PMC310266

[GR278407MONC15] Cokus SJ, Feng S, Zhang X, Chen Z, Merriman B, Haudenschild CD, Pradhan S, Nelson SF, Pellegrini M, Jacobsen SE. 2008. Shotgun bisulphite sequencing of the *Arabidopsis* genome reveals DNA methylation patterning. Nature 452: 215–219. 10.1038/nature06745 18278030 PMC2377394

[GR278407MONC16] Conlin LK, Aref-Eshghi E, McEldrew DA, Luo M, Rajagopalan R. 2022. Long-read sequencing for molecular diagnostics in constitutional genetic disorders. Hum Mutat 43: 1531–1544. 10.1002/humu.24465 36086952 PMC9561063

[GR278407MONC17] Cristiano S, Leal A, Phallen J, Fiksel J, Adleff V, Bruhm DC, Jensen SØ, Medina JE, Hruban C, White JR, 2019. Genome-wide cell-free DNA fragmentation in patients with cancer. Nature 570: 385–389. 10.1038/s41586-019-1272-6 31142840 PMC6774252

[GR278407MONC18] De Coster W, Weissensteiner MH, Sedlazeck FJ. 2021. Towards population-scale long-read sequencing. Nat Rev Genet 22: 572–587. 10.1038/s41576-021-00367-3 34050336 PMC8161719

[GR278407MONC19] Dolzhenko E, van Vugt JJFA, Shaw RJ, Bekritsky MA, van Blitterswijk M, Narzisi G, Ajay SS, Rajan V, Lajoie BR, Johnson NH, 2017. Detection of long repeat expansions from PCR-free whole-genome sequence data. Genome Res 27: 1895–1903. 10.1101/gr.225672.117 28887402 PMC5668946

[GR278407MONC20] Dolzhenko E, English A, Dashnow H, De Sena Brandine G, Mokveld T, Rowell WJ, Karniski C, Kronenberg Z, Danzi MC, Cheung WA, 2024. Characterization and visualization of tandem repeats at genome scale. Nat Biotechnol 42: 1606–1614. 10.1038/s41587-023-02057-3 38168995 PMC11921810

[GR278407MONC21] Ebbert MTW, Farrugia SL, Sens JP, Jansen-West K, Gendron TF, Prudencio M, McLaughlin IJ, Bowman B, Seetin M, DeJesus-Hernandez M, 2018. Long-read sequencing across the *C9orf72* ‘GGGGCC’ repeat expansion: implications for clinical use and genetic discovery efforts in human disease. Mol Neurodegener 13: 46. 10.1186/s13024-018-0274-4 30126445 PMC6102925

[GR278407MONC22] Eggermann T, Perez de Nanclares G, Maher ER, Temple IK, Tümer Z, Monk D, Mackay DJG, Grønskov K, Riccio A, Linglart A, 2015. Imprinting disorders: a group of congenital disorders with overlapping patterns of molecular changes affecting imprinted loci. Clin Epigenetics 7: 123. 10.1186/s13148-015-0143-8 26583054 PMC4650860

[GR278407MONC23] Flusberg BA, Webster DR, Lee JH, Travers KJ, Olivares EC, Clark TA, Korlach J, Turner SW. 2010. Direct detection of DNA methylation during single-molecule, real-time sequencing. Nat Methods 7: 461–465. 10.1038/nmeth.1459 20453866 PMC2879396

[GR278407MONC24] Frommer M, McDonald LE, Millar DS, Collis CM, Watt F, Grigg GW, Molloy PL, Paul CL. 1992. A genomic sequencing protocol that yields a positive display of 5-methylcytosine residues in individual DNA strands. Proc Natl Acad Sci 89: 1827–1831. 10.1073/pnas.89.5.1827 1542678 PMC48546

[GR278407MONC25] Fu Y, Aganezov S, Mahmoud M, Beaulaurier J, Juul S, Treangen TJ, Sedlazeck FJ. 2024. MethPhaser: methylation-based long-read haplotype phasing of human genomes. Nat Commun 15: 5327. 10.1038/s41467-024-49588-0 38909018 PMC11193733

[GR278407MONC26] Füllgrabe J, Gosal WS, Creed P, Liu S, Lumby CK, Morley DJ, Ost TWB, Vilella AJ, Yu S, Bignell H, 2023. Simultaneous sequencing of genetic and epigenetic bases in DNA. Nat Biotechnol 41: 1457–1464. 10.1038/s41587-022-01652-0 36747096 PMC10567558

[GR278407MONC27] Gall-Duncan T, Sato N, Yuen RKC, Pearson CE. 2022. Advancing genomic technologies and clinical awareness accelerates discovery of disease-associated tandem repeat sequences. Genome Res 32: 1–27. 10.1101/gr.269530.120 34965938 PMC8744678

[GR278407MONC28] Genner R, Akeson S, Meredith M, Jerez PA, Malik L, Baker B, Miano-Burkhardt A, CARD-long-read Team, Paten B, Billingsley KJ, 2024. Assessing methylation detection for primary human tissue using Nanopore sequencing. bioRxiv 10.1101/2024.02.29.581569 PMC12047266 40054862

[GR278407MONC29] Gershman A, Sauria MEG, Guitart X, Vollger MR, Hook PW, Hoyt SJ, Jain M, Shumate A, Razaghi R, Koren S, 2022. Epigenetic patterns in a complete human genome. Science 376: eabj5089. 10.1126/science.abj5089 35357915 PMC9170183

[GR278407MONC30] Giesselmann P, Brändl B, Raimondeau E, Bowen R, Rohrandt C, Tandon R, Kretzmer H, Assum G, Galonska C, Siebert R, 2019. Analysis of short tandem repeat expansions and their methylation state with nanopore sequencing. Nat Biotechnol 37: 1478–1481. 10.1038/s41587-019-0293-x 31740840

[GR278407MONC31] Gigante S, Gouil Q, Lucattini A, Keniry A, Beck T, Tinning M, Gordon L, Woodruff C, Speed TP, Blewitt ME, 2019. Using long-read sequencing to detect imprinted DNA methylation. Nucleic Acids Res 47: e46. 10.1093/nar/gkz107 30793194 PMC6486641

[GR278407MONC32] Gilpatrick T, Lee I, Graham JE, Raimondeau E, Bowen R, Heron A, Downs B, Sukumar S, Sedlazeck FJ, Timp W. 2020. Targeted nanopore sequencing with Cas9-guided adapter ligation. Nat Biotechnol 38: 433–438. 10.1038/s41587-020-0407-5 32042167 PMC7145730

[GR278407MONC33] Goenka SD, Gorzynski JE, Shafin K, Fisk DG, Pesout T, Jensen TD, Monlong J, Chang P-C, Baid G, Bernstein JA, 2022. Accelerated identification of disease-causing variants with ultra-rapid nanopore genome sequencing. Nat Biotechnol 40: 1035–1041. 10.1038/s41587-022-01221-5 35347328 PMC9287171

[GR278407MONC34] Gorzynski JE, Goenka SD, Shafin K, Jensen TD, Fisk DG, Grove ME, Spiteri E, Pesout T, Monlong J, Baid G, 2022. Ultrarapid nanopore genome sequencing in a critical care setting. N Engl J Med 386: 700–702. 10.1056/NEJMc2112090 35020984

[GR278407MONC35] Grafodatskaya D, Chung BHY, Butcher DT, Turinsky AL, Goodman SJ, Choufani S, Chen Y-A, Lou Y, Zhao C, Rajendram R, 2013. Multilocus loss of DNA methylation in individuals with mutations in the histone H3 lysine 4 demethylase KDM5C. BMC Med Genomics 6: 1. 10.1186/1755-8794-6-1 23356856 PMC3573947

[GR278407MONC36] Grafodatskaya D, Choufani S, Basran R, Weksberg R. 2017. An update on molecular diagnostic testing of human imprinting disorders. J Pediatr Genet 6: 3–17. 10.1055/s-0036-1593840 28180023 PMC5288000

[GR278407MONC37] Guintivano J, Aryee MJ, Kaminsky ZA. 2013. A cell epigenotype specific model for the correction of brain cellular heterogeneity bias and its application to age, brain region and major depression. Epigenetics 8: 290–302. 10.4161/epi.23924 23426267 PMC3669121

[GR278407MONC38] Guo S, Diep D, Plongthongkum N, Fung H-L, Zhang K, Zhang K. 2017. Identification of methylation haplotype blocks aids in deconvolution of heterogeneous tissue samples and tumor tissue-of-origin mapping from plasma DNA. Nat Genet 49: 635–642. 10.1038/ng.3805 28263317 PMC5374016

[GR278407MONC39] Gustafson JA, Gibson SB, Damaraju N, Zalusky MPG, Hoekzema K, Twesigomwe D, Yang L, Snead AA, Richmond PA, De Coster W, 2024. High-coverage nanopore sequencing of samples from the 1000 Genomes Project to build a comprehensive catalog of human genetic variation. Genome Res 34: 2061–2073. 10.1101/gr.279273.124 39358015 PMC11610458

[GR278407MONC40] Halldorsson BV, Eggertsson HP, Moore KHS, Hauswedell H, Eiriksson O, Ulfarsson MO, Palsson G, Hardarson MT, Oddsson A, Jensson BO, 2022. The sequences of 150,119 genomes in the UK Biobank. Nature 607: 732–740. 10.1038/s41586-022-04965-x 35859178 PMC9329122

[GR278407MONC41] Hansen KD, Langmead B, Irizarry RA. 2012. BSmooth: from whole genome bisulfite sequencing reads to differentially methylated regions. Genome Biol 13: R83. 10.1186/gb-2012-13-10-r83 23034175 PMC3491411

[GR278407MONC42] Hitchins MP. 2015. Constitutional epimutation as a mechanism for cancer causality and heritability? Nat Rev Cancer 15: 625–634. 10.1038/nrc4001 26383139

[GR278407MONC43] Hitchins MP, Wong JJL, Suthers G, Suter CM, Martin DIK, Hawkins NJ, Ward RL. 2007. Inheritance of a cancer-associated *MLH1* germ-line epimutation. N Engl J Med 356: 697–705. 10.1056/NEJMoa064522 17301300

[GR278407MONC44] Höijer I, Tsai Y-C, Clark TA, Kotturi P, Dahl N, Stattin E-L, Bondeson M-L, Feuk L, Gyllensten U, Ameur A. 2018. Detailed analysis of *HTT* repeat elements in human blood using targeted amplification-free long-read sequencing. Hum Mutat 39: 1262–1272. 10.1002/humu.23580 29932473 PMC6175010

[GR278407MONC45] Houseman EA, Accomando WP, Koestler DC, Christensen BC, Marsit CJ, Nelson HH, Wiencke JK, Kelsey KT. 2012. DNA methylation arrays as surrogate measures of cell mixture distribution. BMC Bioinformatics 13: 86. 10.1186/1471-2105-13-86 22568884 PMC3532182

[GR278407MONC46] Hunter JE, Berry-Kravis E, Hipp H, Todd PK. 1998. *FMR1* disorders. In Genereviews® (ed. Adam MP, et al.). University of Washington, Seattle. 20301558

[GR278407MONC47] Ibañez K, Polke J, Hagelstrom RT, Dolzhenko E, Pasko D, Thomas ERA, Daugherty LC, Kasperaviciute D, Smith KR, WGS for Neurological Diseases Group, 2022. Whole genome sequencing for the diagnosis of neurological repeat expansion disorders in the UK: a retrospective diagnostic accuracy and prospective clinical validation study. Lancet Neurol 21: 234–245. 10.1016/S1474-4422(21)00462-2 35182509 PMC8850201

[GR278407MONC48] Irizarry RA, Ladd-Acosta C, Carvalho B, Wu H, Brandenburg SA, Jeddeloh JA, Wen B, Feinberg AP. 2008. Comprehensive high-throughput arrays for relative methylation (CHARM). Genome Res 18: 780–790. 10.1101/gr.7301508 18316654 PMC2336799

[GR278407MONC49] Jaffe AE, Irizarry RA. 2014. Accounting for cellular heterogeneity is critical in epigenome-wide association studies. Genome Biol 15: R31. 10.1186/gb-2014-15-2-r31 24495553 PMC4053810

[GR278407MONC50] Johansson J, Lidéus S, Höijer I, Ameur A, Gudmundsson S, Annerén G, Bondeson M-L, Wilbe M. 2023. A novel quantitative targeted analysis of X-chromosome inactivation (XCI) using nanopore sequencing. Sci Rep 13: 12856. 10.1038/s41598-023-34413-3 37553382 PMC10409790

[GR278407MONC51] Karimzadeh M, Ernst C, Kundaje A, Hoffman MM. 2018. Umap and Bismap: quantifying genome and methylome mappability. Nucleic Acids Res 46: e120. 10.1093/nar/gky677 30169659 PMC6237805

[GR278407MONC52] Katsman E, Orlanski S, Martignano F, Fox-Fisher I, Shemer R, Dor Y, Zick A, Eden A, Petrini I, Conticello SG, 2022. Detecting cell-of-origin and cancer-specific methylation features of cell-free DNA from Nanopore sequencing. Genome Biol 23: 158. 10.1186/s13059-022-02710-1 35841107 PMC9283844

[GR278407MONC53] Kerr L, Kafetzopoulos I, Grima R, Sproul D. 2023. Genome-wide single-molecule analysis of long-read DNA methylation reveals heterogeneous patterns at heterochromatin that reflect nucleosome organisation. PLoS Genet 19: e1010958. 10.1371/journal.pgen.1010958 37782664 PMC10569558

[GR278407MONC54] Khulan B, Thompson RF, Ye K, Fazzari MJ, Suzuki M, Stasiek E, Figueroa ME, Glass JL, Chen Q, Montagna C, 2006. Comparative isoschizomer profiling of cytosine methylation: the HELP assay. Genome Res 16: 1046–1055. 10.1101/gr.5273806 16809668 PMC1524864

[GR278407MONC55] Kinde B, Gabel HW, Gilbert CS, Griffith EC, Greenberg ME. 2015. Reading the unique DNA methylation landscape of the brain: non-CpG methylation, hydroxymethylation, and MeCP2. Proc Natl Acad Sci 112: 6800–6806. 10.1073/pnas.1411269112 25739960 PMC4460470

[GR278407MONC56] Kolmogorov M, Billingsley KJ, Mastoras M, Meredith M, Monlong J, Lorig-Roach R, Asri M, Alvarez Jerez P, Malik L, Dewan R, 2023. Scalable Nanopore sequencing of human genomes provides a comprehensive view of haplotype-resolved variation and methylation. Nat Methods 20: 1483–1492. 10.1038/s41592-023-01993-x 37710018 PMC11222905

[GR278407MONC57] Kong Y, Mead EA, Fang G. 2023. Navigating the pitfalls of mapping DNA and RNA modifications. Nat Rev Genet 24: 363–381. 10.1038/s41576-022-00559-5 36653550 PMC10722219

[GR278407MONC58] LaFlamme CW, Rastin C, Sengupta S, Pennington HE, Russ-Hall SJ, Schneider AL, Bonkowski ES, Almanza Fuerte EP, Allan TJ, Zalusky MP, 2024. Diagnostic utility of DNA methylation analysis in genetically unsolved pediatric epilepsies and CHD2 episignature refinement. Nat Commun 15: 6524. 10.1038/s41467-024-50159-6 39107278 PMC11303402

[GR278407MONC59] Laird PW. 2010. Principles and challenges of genomewide DNA methylation analysis. Nat Rev Genet 11: 191–203. 10.1038/nrg2732 20125086

[GR278407MONC60] Lau BT, Almeda A, Schauer M, McNamara M, Bai X, Meng Q, Partha M, Grimes SM, Lee H, Heestand GM, 2023. Single-molecule methylation profiles of cell-free DNA in cancer with nanopore sequencing. Genome Med 15: 33. 10.1186/s13073-023-01178-3 37138315 PMC10155347

[GR278407MONC61] Lee I, Razaghi R, Gilpatrick T, Molnar M, Gershman A, Sadowski N, Sedlazeck FJ, Hansen KD, Simpson JT, Timp W. 2020. Simultaneous profiling of chromatin accessibility and methylation on human cell lines with nanopore sequencing. Nat Methods 17: 1191–1199. 10.1038/s41592-020-01000-7 33230324 PMC7704922

[GR278407MONC62] Liao W-W, Asri M, Ebler J, Doerr D, Haukness M, Hickey G, Lu S, Lucas JK, Monlong J, Abel HJ, 2023. A draft human pangenome reference. Nature 617: 312–324. 10.1038/s41586-023-05896-x 37165242 PMC10172123

[GR278407MONC63] Lister R, Pelizzola M, Dowen RH, Hawkins RD, Hon G, Tonti-Filippini J, Nery JR, Lee L, Ye Z, Ngo Q-M, 2009. Human DNA methylomes at base resolution show widespread epigenomic differences. Nature 462: 315–322. 10.1038/nature08514 19829295 PMC2857523

[GR278407MONC64] Liu Y, Siejka-Zielińska P, Velikova G, Bi Y, Yuan F, Tomkova M, Bai C, Chen L, Schuster-Böckler B, Song C-X. 2019. Bisulfite-free direct detection of 5-methylcytosine and 5-hydroxymethylcytosine at base resolution. Nat Biotechnol 37: 424–429. 10.1038/s41587-019-0041-2 30804537

[GR278407MONC65] Liu MC, Oxnard GR, Klein EA, Swanton C, Seiden MV, CCGA Consortium. 2020. Sensitive and specific multi-cancer detection and localization using methylation signatures in cell-free DNA. Ann Oncol 31: 745–759. 10.1016/j.annonc.2020.02.011 33506766 PMC8274402

[GR278407MONC66] Logsdon GA, Vollger MR, Eichler EE. 2020. Long-read human genome sequencing and its applications. Nat Rev Genet 21: 597–614. 10.1038/s41576-020-0236-x 32504078 PMC7877196

[GR278407MONC67] Lokk K, Modhukur V, Rajashekar B, Märtens K, Mägi R, Kolde R, Koltšina M, Nilsson TK, Vilo J, Salumets A, 2014. DNA methylome profiling of human tissues identifies global and tissue-specific methylation patterns. Genome Biol 15: r54. 10.1186/gb-2014-15-4-r54 24690455 PMC4053947

[GR278407MONC68] Louis DN, Perry A, Wesseling P, Brat DJ, Cree IA, Figarella-Branger D, Hawkins C, Ng HK, Pfister SM, Reifenberger G, 2021. The 2021 WHO classification of tumors of the central nervous system: a summary. Neuro Oncol 23: 1231–1251. 10.1093/neuonc/noab106 34185076 PMC8328013

[GR278407MONC69] Loyfer N, Magenheim J, Peretz A, Cann G, Bredno J, Klochendler A, Fox-Fisher I, Shabi-Porat S, Hecht M, Pelet T, 2023. A DNA methylation atlas of normal human cell types. Nature 613: 355–364. 10.1038/s41586-022-05580-6 36599988 PMC9811898

[GR278407MONC70] Lyon MF. 1962. Sex chromatin and gene action in the mammalian X-chromosome. Am J Hum Genet 14: 135. 14467629 PMC1932279

[GR278407MONC71] Machado FB, Machado FB, Faria MA, Lovatel VL, da Silva AFA, Radic CP, De Brasi CD, Rios ÁFL, de Sousa Lopes SMC, da Silveira LS, 2014. 5^me^CpG epigenetic marks neighboring a primate-conserved core promoter short tandem repeat indicate X-chromosome inactivation. PLoS One 9: e103714. 10.1371/journal.pone.0103714 25078280 PMC4117532

[GR278407MONC72] Mahmoud M, Doddapaneni H, Timp W, Sedlazeck FJ. 2021. PRINCESS: comprehensive detection of haplotype resolved SNVs, SVs, and methylation. Genome Biol 22: 268. 10.1186/s13059-021-02486-w 34521442 PMC8442460

[GR278407MONC73] Mahmoud M, Harting J, Corbitt H, Chen X, Jhangiani SN, Doddapaneni H, Meng Q, Han T, Lambert C, Zhang S, 2024. Closing the gap: solving complex medically relevant genes at scale. medRxiv 10.1101/2024.03.14.24304179

[GR278407MONC74] Mastrorosa FK, Miller DE, Eichler EE. 2023. Applications of long-read sequencing to Mendelian genetics. Genome Med 15: 42. 10.1186/s13073-023-01194-3 37316925 PMC10266321

[GR278407MONC75] Michels KB, Binder AM, Dedeurwaerder S, Epstein CB, Greally JM, Gut I, Houseman EA, Izzi B, Kelsey KT, Meissner A, 2013. Recommendations for the design and analysis of epigenome-wide association studies. Nat Methods 10: 949–955. 10.1038/nmeth.2632 24076989

[GR278407MONC76] Migeon BR. 2020. X-linked diseases: susceptible females. Genet Med 22: 1156–1174. 10.1038/s41436-020-0779-4 32284538 PMC7332419

[GR278407MONC77] Miller DE, Sulovari A, Wang T, Loucks H, Hoekzema K, Munson KM, Lewis AP, Fuerte EPA, Paschal CR, Walsh T, 2021. Targeted long-read sequencing identifies missing disease-causing variation. Am J Hum Genet 108: 1436–1449. 10.1016/j.ajhg.2021.06.006 34216551 PMC8387463

[GR278407MONC78] Miller DE, Hanna P, Galey M, Reyes M, Linglart A, Eichler EE, Jüppner H. 2022. Targeted long-read sequencing identifies a retrotransposon insertion as a cause of altered *GNAS* exon A/B methylation in a family with autosomal dominant pseudohypoparathyroidism type 1b (PHP1B). J Bone Miner Res 37: 1711–1719. 10.1002/jbmr.4647 35811283 PMC9474630

[GR278407MONC79] Min JL, Hemani G, Hannon E, Dekkers KF, Castillo-Fernandez J, Luijk R, Carnero-Montoro E, Lawson DJ, Burrows K, Suderman M, 2021. Genomic and phenotypic insights from an atlas of genetic effects on DNA methylation. Nat Genet 53: 1311–1321. 10.1038/s41588-021-00923-x 34493871 PMC7612069

[GR278407MONC80] Monk D, Mackay DJG, Eggermann T, Maher ER, Riccio A. 2019. Genomic imprinting disorders: lessons on how genome, epigenome and environment interact. Nat Rev Genet 20: 235–248. 10.1038/s41576-018-0092-0 30647469

[GR278407MONC81] Montaño CM, Irizarry RA, Kaufmann WE, Talbot K, Gur RE, Feinberg AP, Taub MA. 2013. Measuring cell-type specific differential methylation in human brain tissue. Genome Biol 14: R94. 10.1186/gb-2013-14-8-r94 24000956 PMC4054676

[GR278407MONC82] Moss J, Magenheim J, Neiman D, Zemmour H, Loyfer N, Korach A, Samet Y, Maoz M, Druid H, Arner P, 2018. Comprehensive human cell-type methylation atlas reveals origins of circulating cell-free DNA in health and disease. Nat Commun 9: 5068. 10.1038/s41467-018-07466-6 30498206 PMC6265251

[GR278407MONC83] Nakamura W, Hirata M, Oda S, Chiba K, Okada A, Mateos RN, Sugawa M, Iida N, Ushiama M, Tanabe N, 2024. Assessing the efficacy of target adaptive sampling long-read sequencing through hereditary cancer patient genomes. NPJ Genom Med 9: 11. 10.1038/s41525-024-00394-z 38368425 PMC10874402

[GR278407MONC84] Nanda AS, Wu K, Irkliyenko I, Woo B, Ostrowski MS, Clugston AS, Sayles LC, Xu L, Satpathy AT, Nguyen HG, 2024. Direct transposition of native DNA for sensitive multimodal single-molecule sequencing. Nat Genet 56: 1300–1309. 10.1038/s41588-024-01748-0 38724748 PMC11176058

[GR278407MONC85] Ni P, Nie F, Zhong Z, Xu J, Huang N, Zhang J, Zhao H, Zou Y, Huang Y, Li J, 2023. DNA 5-methylcytosine detection and methylation phasing using PacBio circular consensus sequencing. Nat Commun 14: 4054. 10.1038/s41467-023-39784-9 37422489 PMC10329642

[GR278407MONC86] O'Neill K, Pleasance E, Fan J, Akbari V, Chang G, Dixon K, Csizmok V, MacLennan S, Porter VL, Galbraith A, 2024. Long-read sequencing of an advanced cancer cohort resolves rearrangements, unravels haplotypes, and reveals methylation landscapes. Cell Genom 4: 100674. 10.1016/j.xgen.2024.100674 39406235 PMC11605692

[GR278407MONC87] Paschal CR, Zalusky MPG, Beck AE, Gillentine MA, Narayanan J, Damaraju N, Goffena J, Storz SHR, Miller DE. 2025. Concordance of whole-genome long-read sequencing with standard clinical testing for Prader-Willi and Angelman syndromes. J Mol Diagn 10.1016/j.jmoldx.2024.12.003 PMC11881775 39756651

[GR278407MONC88] Pretto DI, Mendoza-Morales G, Lo J, Cao R, Hadd A, Latham GJ, Durbin-Johnson B, Hagerman R, Tassone F. 2014. CGG allele size somatic mosaicism and methylation in *FMR1* premutation alleles. J Med Genet 51: 309–318. 10.1136/jmedgenet-2013-102021 24591415 PMC4010431

[GR278407MONC89] Rakyan VK, Down TA, Thorne NP, Flicek P, Kulesha E, Gräf S, Tomazou EM, Bäckdahl L, Johnson N, Herberth M, 2008. An integrated resource for genome-wide identification and analysis of human tissue-specific differentially methylated regions (tDMRs). Genome Res 18: 1518–1529. 10.1101/gr.077479.108 18577705 PMC2527707

[GR278407MONC90] Rasmussen A, Hildonen M, Vissing J, Duno M, Tümer Z, Birkedal U. 2022. High resolution analysis of *DMPK* hypermethylation and repeat interruptions in myotonic dystrophy type 1. Genes (Basel) 13: 970. 10.3390/genes13060970 35741732 PMC9222588

[GR278407MONC91] Rausch T, Snajder R, Leger A, Simovic M, Giurgiu M, Villacorta L, Henssen AG, Fröhling S, Stegle O, Birney E, 2023. Long-read sequencing of diagnosis and post-therapy medulloblastoma reveals complex rearrangement patterns and epigenetic signatures. Cell Genom 3: 100281. 10.1016/j.xgen.2023.100281 37082141 PMC10112291

[GR278407MONC92] Richards S, Aziz N, Bale S, Bick D, Das S, Gastier-Foster J, Grody WW, Hegde M, Lyon E, Spector E, 2015. Standards and guidelines for the interpretation of sequence variants: a joint consensus recommendation of the American College of Medical Genetics and Genomics and the Association for Molecular Pathology. Genet Med 17: 405–424. 10.1038/gim.2015.30 25741868 PMC4544753

[GR278407MONC93] Rizzardi LF, Hickey PF, Rodriguez DiBlasi V, Tryggvadóttir R, Callahan CM, Idrizi A, Hansen KD, Feinberg AP. 2019. Neuronal brain-region-specific DNA methylation and chromatin accessibility are associated with neuropsychiatric trait heritability. Nat Neurosci 22: 307–316. 10.1038/s41593-018-0297-8 30643296 PMC6348048

[GR278407MONC94] Sadikovic B, Levy MA, Aref-Eshghi E. 2020. Functional annotation of genomic variation: DNA methylation episignatures in neurodevelopmental Mendelian disorders. Hum Mol Genet 29: R27–R32. 10.1093/hmg/ddaa144 32644126

[GR278407MONC95] Sanchez-Delgado M, Riccio A, Eggermann T, Maher ER, Lapunzina P, Mackay D, Monk D. 2016. Causes and consequences of multi-locus imprinting disturbances in humans. Trends Genet 32: 444–455. 10.1016/j.tig.2016.05.001 27235113

[GR278407MONC96] Shen SY, Singhania R, Fehringer G, Chakravarthy A, Roehrl MHA, Chadwick D, Zuzarte PC, Borgida A, Wang TT, Li T, 2018. Sensitive tumour detection and classification using plasma cell-free DNA methylomes. Nature 563: 579–583. 10.1038/s41586-018-0703-0 30429608

[GR278407MONC97] Sigurpalsdottir BD, Stefansson OA, Holley G, Beyter D, Zink F, Hardarson MÞ, Sverrisson SÞ, Kristinsdottir N, Magnusdottir DN, Magnusson OÞ, 2024. A comparison of methods for detecting DNA methylation from long-read sequencing of human genomes. Genome Biol 25: 69. 10.1186/s13059-024-03207-9 38468278 PMC10929077

[GR278407MONC98] Simpson JT, Workman RE, Zuzarte PC, David M, Dursi LJ, Timp W. 2017. Detecting DNA cytosine methylation using nanopore sequencing. Nat Methods 14: 407–410. 10.1038/nmeth.4184 28218898

[GR278407MONC99] Spector E, Behlmann A, Kronquist K, Rose NC, Lyon E, Reddi HV, ACMG Laboratory Quality Assurance Committee. 2021. Laboratory testing for fragile X, 2021 revision: a technical standard of the American College of Medical Genetics and Genomics (ACMG). Genet Med 23: 799–812. 10.1038/s41436-021-01115-y 33795824

[GR278407MONC0100] Stefansson OA, Sigurpalsdottir BD, Rognvaldsson S, Halldorsson GH, Juliusson K, Sveinbjornsson G, Gunnarsson B, Beyter D, Jonsson H, Gudjonsson SA, 2024. The correlation between CpG methylation and gene expression is driven by sequence variants. Nat Genet 56: 1624–1631. 10.1038/s41588-024-01851-2 39048797 PMC11319203

[GR278407MONC100] Stergachis AB, Debo BM, Haugen E, Churchman LS, Stamatoyannopoulos JA. 2020. Single-molecule regulatory architectures captured by chromatin fiber sequencing. Science 368: 1449–1454. 10.1126/science.aaz1646 32587015

[GR278407MONC101] Stevanovski I, Chintalaphani SR, Gamaarachchi H, Ferguson JM, Pineda SS, Scriba CK, Tchan M, Fung V, Ng K, Cortese A, 2022. Comprehensive genetic diagnosis of tandem repeat expansion disorders with programmable targeted nanopore sequencing. Sci Adv 8: eabm5386. 10.1126/sciadv.abm5386 35245110 PMC8896783

[GR278407MONC102] Sun K, Jiang P, Chan KCA, Wong J, Cheng YKY, Liang RHS, Chan W-K, Ma ESK, Chan SL, Cheng SH, 2015. Plasma DNA tissue mapping by genome-wide methylation sequencing for noninvasive prenatal, cancer, and transplantation assessments. Proc Natl Acad Sci 112: E5503–E5512. 10.1073/pnas.1508736112 26392541 PMC4603482

[GR278407MONC103] Sun Z, Vaisvila R, Hussong L-M, Yan B, Baum C, Saleh L, Samaranayake M, Guan S, Dai N, Corrêa IR Jr, 2021. Nondestructive enzymatic deamination enables single-molecule long-read amplicon sequencing for the determination of 5-methylcytosine and 5-hydroxymethylcytosine at single-base resolution. Genome Res 31: 291–300. 10.1101/gr.265306.120 33468551 PMC7849414

[GR278407MONC104] Tak Y, Schneider A, Santos E, Randol JL, Tassone F, Hagerman P, Hagerman RJ. 2024. Unmethylated mosaic full mutation males without fragile X syndrome. Genes (Basel) 15: 331. 10.3390/genes15030331 38540390 PMC10970065

[GR278407MONC105] Tsai Y-C, Greenberg D, Powell J, Höijer I, Ameur A, Strahl M, Ellis E, Jonasson I, Pinto RM, Wheeler VC, 2017. Amplification-free, CRISPR-Cas9 targeted enrichment and SMRT sequencing of repeat-expansion disease causative genomic regions. bioRxiv 10.1101/203919

[GR278407MONC106] Unterman I, Avrahami D, Katsman E, Triche TJ Jr, Glaser B, Berman BP. 2024. CelFiE-ISH: a probabilistic model for multi-cell type deconvolution from single-molecule DNA methylation haplotypes. Genome Biol 25: 151. 10.1186/s13059-024-03275-x 38858759 PMC11163775

[GR278407MONC107] Vaisvila R, Ponnaluri VKC, Sun Z, Langhorst BW, Saleh L, Guan S, Dai N, Campbell MA, Sexton BS, Marks K, 2021. Enzymatic methyl sequencing detects DNA methylation at single-base resolution from picograms of DNA. Genome Res 31: 1280–1289. 10.1101/gr.266551.120 34140313 PMC8256858

[GR278407MONC108] Vollger MR, Korlach J, Eldred KC, Swanson E, Underwood JG, Bohaczuk SC, Mao Y, Cheng YH, Ranchalis J, Blue EE, 2025. Synchronized long-read genome, methylome, epigenome and transcriptome profiling resolve a Mendelian condition. Nat Genet 10.1038/s41588-024-02067-0 PMC12077378 39880924

[GR278407MONC109] Weber M, Davies JJ, Wittig D, Oakeley EJ, Haase M, Lam WL, Schübeler D. 2005. Chromosome-wide and promoter-specific analyses identify sites of differential DNA methylation in normal and transformed human cells. Nat Genet 37: 853–862. 10.1038/ng1598 16007088

[GR278407MONC110] Wu Z, Abdullaev Z, Pratt D, Chung H-J, Skarshaug S, Zgonc V, Perry C, Pack S, Saidkhodjaeva L, Nagaraj S, 2022. Impact of the methylation classifier and ancillary methods on CNS tumor diagnostics. Neuro Oncol 24: 571–581. 10.1093/neuonc/noab227 34555175 PMC8972234

[GR278407MONC111] Yamada M, Okuno H, Okamoto N, Suzuki H, Miya F, Takenouchi T, Kosaki K. 2023. Diagnosis of Prader-Willi syndrome and Angelman syndrome by targeted nanopore long-read sequencing. Eur J Med Genet 66: 104690. 10.1016/j.ejmg.2022.104690 36587803

[GR278407MONC112] Yu SCY, Jiang P, Peng W, Cheng SH, Cheung YTT, Tse OYO, Shang H, Poon LC, Leung TY, Chan KCA, 2021. Single-molecule sequencing reveals a large population of long cell-free DNA molecules in maternal plasma. Proc Natl Acad Sci 118: e2114937118. 10.1073/pnas.2114937118 34873045 PMC8685924

[GR278407MONC113] Yu SCY, Choy LYL, Lo YMD. 2023a. “Longing” for the next generation of liquid biopsy: the diagnostic potential of long cell-free DNA in oncology and prenatal testing. Mol Diagn Ther 27: 563–571. 10.1007/s40291-023-00661-2 37474843 PMC10435595

[GR278407MONC114] Yu SCY, Deng J, Qiao R, Cheng SH, Peng W, Lau SL, Choy LYL, Leung TY, Wong J, Wong VW-S, 2023b. Comparison of single molecule, real-time sequencing and nanopore sequencing for analysis of the size, end-motif, and tissue-of-origin of long cell-free DNA in plasma. Clin Chem 69: 168–179. 10.1093/clinchem/hvac180 36322427

[GR278407MONC115] Zhong J-Y, Niu L, Lin Z-B, Bai X, Chen Y, Luo F, Hou C, Xiao C-L. 2023. High-throughput Pore-C reveals the single-allele topology and cell type-specificity of 3D genome folding. Nat Commun 14: 1250. 10.1038/s41467-023-36899-x 36878904 PMC9988853

[GR278407MONC116] Ziller MJ, Hansen KD, Meissner A, Aryee MJ. 2015. Coverage recommendations for methylation analysis by whole-genome bisulfite sequencing. Nat Methods 12: 230–232. 10.1038/nmeth.3152 25362363 PMC4344394

